# Interventions Delivered in the Community Pharmacy to Manage Allergic Rhinitis- A Systematic Review of the Literature

**DOI:** 10.3390/pharmacy8020080

**Published:** 2020-05-06

**Authors:** Jéssica José, Biljana Cvetkovski, Vicky Kritikos, Rachel Tan, Sinthia Bosnic-Anticevich, Olga Lourenço

**Affiliations:** 1 Faculty of Health Sciences and CICS—UBI, Health Sciences Research Centre, University of Beira Interior, 6200-506 Covilhã, Portugal; jdajose@hotmail.com; 2 Woolcock Institute of Medical Research, University of Sidney and Sydney Local Health District, Glebe 2037, Australia; biljana.cvetkovski@sydney.edu.au (B.C.); vicky.kritikos@sydney.edu.au (V.K.); rachel.sze.tan@sydney.edu.au (R.T.); sinthia.bosnic-anticevich@sydney.edu.au (S.B.-A.)

**Keywords:** allergic rhinitis, community pharmacy, pharmacist

## Abstract

Pharmacists have a valuable role in the management of allergic rhinitis (AR) at the community pharmacy level. This role has been reported extensively in numerous papers. However, a systematic review of the available literature and a comprehensive analysis of the outcomes has not been published. This systematic review aimed to evaluate the impact of interventions developed by pharmacists on clinical AR outcomes. A thorough search was performed in three electronic databases, including studies published between January 2000 and June 2019. After the selection process, only three articles met the inclusion criteria and were further analysed. Despite the scarcity of the available studies, in all of them was clear that the pharmacist plays a pivotal role in the management of AR, significantly improving the patients’ quality of life and symptom control. This systematic review also stresses the utmost importance to investigate and report practices and interventions developed by pharmacists using measurable outcomes.

## 1. Introduction

Allergic rhinitis (AR), commonly referred to as hayfever, is a chronic upper respiratory condition resulting from inflammation of the nasal mucosa. The common symptoms of AR include sneezing, rhinorrhoea and nasal congestion, induced by an immunological response following exposure to allergens such as pollen, house dust mites, moulds and animal dander in sensitised individuals [1]. While AR is often portrayed as a nuisance or trivial condition, the reality is that uncontrolled AR is a disabling and intrusive disease: disabling in terms of its chronicity, and intrusive in terms of its symptom burden and impact on patients’ lives and the lives of their families. Poorly controlled AR can have a substantial negative impact on a patient’s quality of life, including impairments in concentration, work productivity, social interactions and sleep. It is a significant cause of morbidity and imposes a high socioeconomic burden due to the direct treatment costs and the indirect costs due to absenteeism from the workplace and reduced productivity at work [2,3,4]. The impact of poorly controlled AR can also extend into co-existing asthma, where it can worsen asthma symptom control and increase the risk of exacerbations or flare-ups [5].

The socioeconomic and health burden of poorly controlled AR on individuals and society can be minimised with optimal AR management strategies, that encompass patients education, including allergen minimisation strategies, pharmacotherapy and the addition of allergen-specific immunotherapy in severe cases of AR [6]. AR can be optimally managed in the primary care setting; however, there are several challenges encountered by both general practitioners (GPs) and community pharmacists [7,8]. For GPs, AR is becoming more challenging to diagnose, and management is often complicated by polysensitization and the presence of both allergic and non-allergic disease components. For community pharmacists, the fact that the majority of patients who present to community pharmacy have “self-diagnosed” their condition [9,10,11,12,13] and/or self-select over-the-counter AR treatments in a community pharmacy, without seeking pharmacist advice are major challenges [7,8,14,15].

The non-governmental organization Allergic Rhinitis and its Impact on Asthma (ARIA) issued specific guidelines for the management of AR in the community pharmacy, first in 2004 [14] with a recent update [16]. Other guidelines have also been issued by other scientific societies, including the Standards of Care Committee of the British Society of Allergy and Clinical Immunology, albeit without specific recommendations to pharmacists [17]. While pharmacists cannot confirm a diagnosis of AR, they have a key role to play in the management of AR, by ensuring their patients have received a diagnosis, are guided to optimal treatment for their nasal symptoms, and/or are referred to their GPs if needed.

Pharmacists can interview patients and determine whether they require a referral and further medical investigation [16]. They play a critical role in engaging patients who self-select over-the-counter AR medication, which is often associated with suboptimal management [9,13,18,19,20,21]. In a study by Lourenço et al. (2014), uncontrolled AR was identified in 87% of pharmacy patients [22]. This was further confirmed in a recent Australian study where almost 85% of people who self-selected their AR medication, made a suboptimal choice [13]. For patients who self-manage their AR and bypass health care professionals (HCPs), it’s of the utmost importance to recapture their attention. Since patients have reported that they discuss their medication with their pharmacist more often than with their physician [18], the role of the pharmacist in AR management needs to be promoted. Within the context of health care delivery, the role of the pharmacist includes the provision of medication counselling and disease education, monitoring of treatment response, provision of lifestyle recommendation and establishment of therapeutic goals [16,23]. The provision of pharmacist counselling and their interactions with patients have been shown to reduce the likelihood of experiencing medication adverse effects [24], increase adherence to therapy [25], and improve health outcomes [26]. It is crucial for pharmacists to harness these skills and incorporate them into AR management to identify and optimise poorly controlled AR.

While there is a large body of literature available regarding the management of AR within the community pharmacy setting, to date, no systematic review has evaluated community pharmacists’ intervention in AR management. This systematic review aims to evaluate the impact of community pharmacists’ interventions on clinical AR outcomes among adult patients.

## 2. Materials and Methods

### 2.1. Data Sources and Searches

This systematic review was performed according to the methodology recommended by the PRISMA guidelines [27] from January 2018 to June 2019. The research was conducted using three electronic databases: PubMed, Web of Science and Cochrane Central Register of Controlled Trials. Table 1 shows the specific research strategy adopted; the keywords that were used included MeSH and general terms relating to pharmacy, pharmacists and AR (“pharmacy” [MeSH], “community pharmacy”, “pharmaceutical services” [MeSH], “pharmaceutical care”, “pharmacist”, “clinical pharmacy”, “allergic rhinitis” [MeSH] and “hay fever”).

### 2.2. Study Selection

To be included in the systematic review, the studies had to meet the following inclusion criteria: (1) published between January 2000 and June 2019; (2) written in Portuguese, English, Spanish or French; (3) original article/investigation (primary literature); and (4) report the results of community pharmacists interventions in AR. All articles that did not meet the mentioned inclusion criteria were not considered. No limitation regarding the age of the participants was considered. References obtained from different databases were compared to identify and remove duplicates.

### 2.3. Data Synthesis

The titles and abstracts of all articles obtained from the database search were screened to identify potentially relevant articles (which fulfilled the inclusion criteria). Non-relevant articles were deleted while potentially relevant articles identified were obtained. The full text of the eligible studies was read and evaluated, considering the objectives of this review. All non-original and non-experimental studies that did not report the clinical results from the implemented intervention or interventions that were not exclusively implemented by community pharmacists were also excluded. Additionally, potentially relevant studies in the bibliographic references of the selected studies of interest were searched. The studies identified were reviewed by two investigators (J.J. and O.L.).

The relevant information that was extracted from each eligible article, was (1) the source (author and year of publication), (2) the country where the intervention was performed, (3) the objective of the intervention, (4) the type of study (5) the outcomes assessed, (6) the tools used for outcome assessment, (7) the number of participants (patients with AR, pharmacists and pharmacies), (8) the intervention results, and (9) study limitations.

### 2.4. Quality Assessment

For assessment of study quality and risk of bias, we applied the ‘Risk of Bias’ tool described in the Cochrane Review Group Handbook and the STROBE statement checklists (available on https://www.strobe-statement.org).

## 3. Results

**Figure 1:**
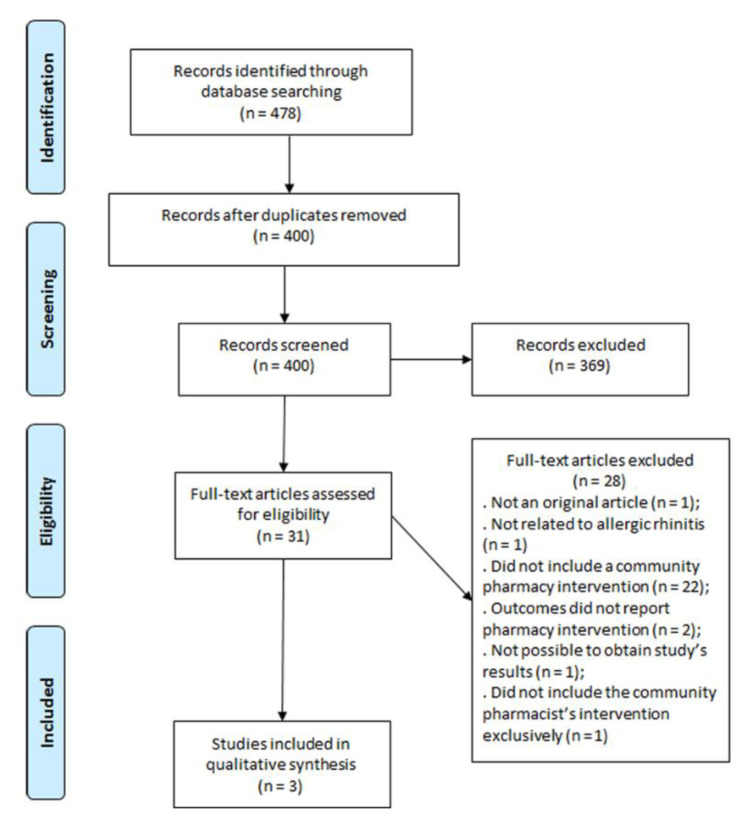
Selection of the studies to be included in the systematic review.

Electronic searching from the different databases resulted in a total of 478 citations, of which 78 duplicates were removed. Their titles and abstracts were then read to assess their agreement with the initially defined inclusion criteria, which resulted in 31 studies. Of these, 28 were excluded, which resulted in three studies included in the systematic review. This process is illustrated in Figure 1.

The three intervention studies [10,28,29] that were included evaluated the improvement of patients’ quality of life (QoL) after intervention by community pharmacists. This assessment was performed using validated tools, namely the Mini Rhinoconjunctivitis Quality of Life Questionnaire (Mini RQLQ ©), the Visual Analog Scales (VAS) [10,28] and also the SF-12v2 Health Survey [29] (validated questionnaire for Bulgaria). Table 2 summarises the information extracted from each study included in this systematic review.

The intervention study conducted by Arsoy et al. focused on AR education, including avoidance of allergen exposure, counselling on medication and training on the administration technique of nasal medication [10]. The study involved two visits: baseline and 6 weeks post-baseline for each patient.

The study conducted by O’Connor et al. [28] compared two interventions developed by community pharmacists. In the first, participants defined their relevant goals and strategies relating to their AR; in the second, participants had their goals and strategies defined by a pharmacist. Although pharmacies were randomised, the study did not have a control group per se, as both groups had support from community pharmacists. For each patient, the intervention involved a series of three visits: baseline, one-week form baseline and 6 weeks from baseline.

Both studies used the Mini RQLQ© to assess the QoL of the patients before and after the interventions.

The Mini RQLQ© scores were significantly reduced between the pharmacy visits resulting in statistically significant QoL improvements [10,28]. However, the study conducted by O’Connor, J. et al. reported that there was no significant improvement between the pharmacist-defined goals group and the patient-defined goals group (F (2.35) = 1.03, p > 0.05).

The study by Todorova et al. [29] explored the impact of pharmaceutical care and patient counselling on QoL, as measured by the SF-12v2 Health Survey. The baseline survey involved patients with pronounced AR symptoms seeking medical advice in the pharmacy. A follow-up survey of the patients QoL was performed after the intervention and dispensing of the appropriate OTC product according to ARIA guidelines (no period between visits is referred). The study showed an improvement in patients’ QoL in various aspects (physical and mental health and social functions).

Regarding the severity of symptoms, both the Arsoy G. et al. [10] and O’Connor, J. et al. [28] studies achieved a significant decrease in the severity of symptoms VAS scores. However, the former reported a decrease in both groups (intervention and control) [10] while in the latter a greater statistically significant decrease was reported in the group where pharmacists set patients’ targets [28].

## 4. Discussion and Conclusions

This systematic review highlights the paucity of research exploring pharmacist interventions in AR management and draws to attention the urgency of the requirement for further reseach in this field, especially given the burden caused by sub-optimal management of AR internationally [3,30]. Although it is difficult to evaluate the impact of community pharmacists’ interventions on clinical AR outcomes among adult patients given the limited amount of studies identified as a part of this review, it is with certainty that we can say the the role of the pharmacist in AR management has been underoptimised thus far. In the current health care environment where AR is frequently undertreated, underdiagnosed and patient self-selection is profound, the role of the pharmacist is more important than ever [31,32].

A major limitation of this review was the scarcity of intervention studies in AR in the community pharmacy setting, as well as the limited quality of existing studies. The most profound limitation across all the three studies was the limited number of participants, both pharmacists and patients. Similarly, the studies were conducted were confined to specific geographical areas and not necessarily applicable to wider health care settings international. O’Connor et al., also has limitations of not having a control group and focussing on OAH use, whose overuse is currently associated with poorer AR outcomes and is only recommended in the most mildest of AR cases [16,33].

However, despite the limited number of pharmacy interventions evaluated, this review has shown that pharmacists play a crucial role in helping patients minimise their AR symptoms, providing information and advice on treatment and monitoring outcomes. From these studies it can be concluded that interventions by community pharmacists in the management of AR can significantly improve patients’ QoL while also improving disease control; however, further research in this field is needed. Further interventions are imperative to fully understand the pharmacist’s role in the management of AR since the community pharmacy is the venue of choice for patients to receive counselling regarding AR and often the only place where patients seek healthcare for their AR.

Patients with AR have needs that are not being met by current practices, e.g., diagnosis is absent in many cases, and there is inadequate counselling and treatment selection by pharmacists. These facts may be related to pharmacists’ lack of knowledge about ARIA guidelines [11,34] and ultimately lead to lack of disease control. These unmet needs are opportunities for pharmacists to intervene in order to improve patient’s QoL and disease management, thereby reducing the burden of AR.

## Figures and Tables

**Table 1:** Search strategy adopted for this systematic review.

	PubMed		Web of Science		Cochrane Central Register of Controlled Trials	
	Search	Result	Search	Result	Search	Result
**1**	Pharmacy	369,988	TOPIC:(Pharmacy)	39,661	Pharmacy	12,793
**2**	Community pharmacy	25,790	TOPIC:(Community Pharmacy)	7911	Community pharmacy	1619
**3**	Pharmaceutical services	75,375	TOPIC:(Pharmaceutical services)	5499	Pharmaceutical services	1332
**4**	Pharmaceutical care	94,491	TOPIC:(Pharmaceutical care)	16,051	Pharmaceutical care	4205
**5**	Pharmacist *	33,612	TOPIC:(Pharmacist)	30,192	Pharmacist *	3914
**6**	Clinical pharmacy	87,664	TOPIC:(Clinical Pharmacy)	9309	Clinical pharmacy	10,802
**7**	1 OR 2 OR 3 OR 4 OR 5 OR 6	439,230	1 OR 2 OR 3 OR 4 OR 5 OR 6	74,702	1 OR 2 OR 3 OR 4 OR 5 OR 6	17,638
**8**	Allergic rhinitis	29,036	TOPIC:(Allergic Rhinitis)	22,524	Allergic rhinitis	6743
**9**	Hay fever	15,255	TOPIC:(Hay fever)	4196	Hay fever	714
**10**	8 OR 9	30,080	8 OR 9	25,647	8 OR 9	6977
**11**	7 AND 10	260	7 AND 10	117	7 AND 10	33

* a PubMed notation to indicate that the search included “pharmacist” and “pharmacists” as seach keywords.

**Table 2:** Summary of studies that met the inclusion criteria and were included in the systematic review.

Author & Year of Publication	Country	Objective	Type of Study	Outcomes	Tools used to Evaluate the Outcomes	Number of Participants	Results	Limitations
*Arsoy, G.* et al. (2018) [10]	Cyprus (Northern Cyprus)	Evaluate the effectiveness of educational intervention by pharmacists to improve the care of patients with AR	Randomized controlled trial (RCT)	Quality of life (QoL) Symptom severity	Mini Rhinoconjunctivitis Quality of Life Questionnaire (Mini RQLQ©) Analog visual scales (VAS)	Pharmacists (n = 70) Patients (n = 63) *	The pharmaceutical intervention significantly improved the quality of life of patients. Both groups experienced an improvement in symptom severity.	Small number of pharmacists and patients
*O’ Connor, J.* et al. (2007) [28]	Australia (Sydney)	Examine the impact of community pharmacist goal setting on AR management versus patient setting	Mixed method parallel-group study, with randomization of participating pharmacies	Quality of life (QoL) Symptom severity	Mini Rhinoconjunctivitis Quality of Life Questionnaire (Mini RQLQ©) Analog visual scales (VAS)	Pharmacists (n = 8) Patients (n = 47) #	The intervention group showed greater improvements in symptom severity than the control group.	Small number of pharmacists and patients, only metropolitan Sydney community pharmacies
*Todorova, A.* et al. (2017) [29]	Bulgaria (Varna)	Assess the impact of pharmaceutical care on AR management	Case study	Quality of life (QoL)	SF-12v2 Health Survey	Patients (n = 63)	The pharmaceutical intervention significantly improved the quality of life of patients with AR	Small number of patients, only city of Varna

* In total, 32 patients in the intervention group and 31 in the control group; # 26 patients in the patient-defined goals group and 21 patients in the pharmacist-defined goals group.
